# Basroparib inhibits YAP‐driven cancers by stabilizing angiomotin

**DOI:** 10.1002/1878-0261.70209

**Published:** 2026-01-22

**Authors:** Young‐Ju Kwon, Dong Young Kim, Yuna Kim, Uk‐Il Kim, Jae‐Sung Kim

**Affiliations:** ^1^ Division of Radiation Biomedical Research Korea Institute of Radiological and Medical Sciences Seoul Korea; ^2^ Radiological and Medico‐Oncological Sciences University of Science and Technology Seoul Korea; ^3^ ST Pharm Co., Ltd. Seoul Korea

**Keywords:** angiomotin, basroparib, MEK inhibitor resistance, tankyrase inhibitor, YAP

## Abstract

Yes‐associated protein (YAP) is a key oncogenic effector and a well‐established driver of resistance to anticancer therapies, especially in tumors harboring KRAS mutations. Although YAP is clinically relevant, drug‐development efforts that directly inhibit its activity have been limited. Here, we show that basroparib—a selective tankyrase (TNKS) inhibitor that suppresses Wnt signaling—attenuates YAP‐driven oncogenic programs by stabilizing angiomotin (AMOT), an endogenous negative regulator of YAP. In colorectal cancer (CRC) cells, basroparib increased AMOT protein abundance, promoted AMOT–YAP complex formation, and enforced cytoplasmic sequestration of YAP, thereby dampening YAP‐dependent transcription. Basroparib preferentially sensitized YAP‐overexpressing, KRAS‐mutant CRC cell lines to MEK inhibition by inhibiting YAP signaling. In MEK inhibitor‐resistant CRC models, in which elevated YAP activity mediates escape, basroparib restored drug sensitivity both *in vitro* and *in vivo*. The compound also enhanced MEK inhibitor efficacy in other YAP‐active tumor types, while exerting minimal effects in YAP‐inactive models. Taken together, these results identify basroparib—now progressing through clinical development (Phase I, NCT04505839)—as a promising agent for dual Wnt–YAP pathway blockade and for overcoming therapeutic resistance in YAP‐driven cancers.

## Graphical abstract

Basroparib, a selective tankyrase inhibitor, suppresses Wnt signaling and attenuates YAP‐driven oncogenic programs by stabilizing angiomotin. It promotes AMOT–YAP complex formation, enforces cytoplasmic YAP sequestration, inhibits YAP/TEAD transcription, and sensitizes YAP‐active cancers, including KRAS‐mutant colorectal cancer, to MEK inhibition.

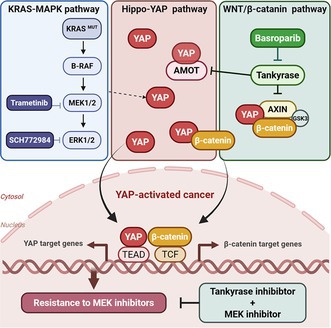

**Abbreviations**
AMOT: angiomotin
Bas: basroparib
CHX: cycloheximide
CI: combination index
CRC: colorectal cancer
CTGF: connective tissue growth factor
CYR61: cysteine‐rich angiogenic inducer 61
ERK: extracellular signal‐regulated kinase
FBS: fetal bovine serum
KRAS: kirsten rat sarcoma
MEK: mitogen‐activated protein kinase kinase
ns: not significant
PO: Per OS (by mouth)
PI3K: phosphoinositide 3‐kinase
QD: Quaque die (daily)
SAV1: salvador homolog 1
SCH: SCH772984
TAZ: transcriptional coactivator with PDZ‐binding motif
TEAD4: transcriptional enhancer activator domain4
TGI: tumor growth inhibition
TNKS: tankyrase
Tra: trametinib
Veh: vehicle
WT: wild‐type
YAP: yes‐associated protein
CDK2/4/6: cyclin‐dependent kinase2/4/6
AMOTL1/2: angiomotin like 1/2
LATS1/2: large tumor suppressor1/2
MST1/2: mammalian STE20‐like protein kinase 1/2

## 1 Introduction

Yes‐associated protein (YAP), a key transcriptional coactivator within the Hippo signaling pathway, plays a pivotal role in tumor progression and resistance to targeted therapies, including MEK inhibitors [1, 2, 3]. As a core effector of the Hippo pathway, YAP is frequently upregulated in various cancers, where it promotes oncogenesis by regulating genes involved in cell proliferation, apoptosis, and stemness [3]. Under physiological conditions, the Hippo pathway—comprising MST1/2, SAV1, LATS1/2, YAP, and its paralog TAZ—maintains tissue homeostasis by balancing cell growth and cell death. Dysregulation of this pathway leads to sustained YAP activation and malignant transformation [1]. Recent studies have highlighted the therapeutic potential of YAP inhibition, particularly for overcoming resistance in KRAS‐mutant cancers that are refractory to MEK inhibition [1, 2, 3]. YAP blockade has been shown to resensitize MEK inhibitor–resistant cancer cells across multiple tumor types, including colorectal, lung, neuroblastoma, melanoma, and head and neck cancers [2, 4], underscoring the clinical relevance of targeting YAP in resistant malignancies.

In colorectal cancer (CRC), YAP signaling is tightly integrated with the Wnt/β‐catenin pathway, another critical regulator of proliferation, differentiation, and cancer stemness [5, 6, 7]. Activation of Wnt signaling leads to the disruption of the β‐catenin destruction complex, resulting in nuclear accumulation of β‐catenin and transcriptional activation of oncogenic targets. Concurrently, nuclear YAP enhances TEAD‐dependent gene expression and cooperates with β‐catenin/TCF complexes to amplify the transcription of stemness‐related genes such as LGR5, CCND1, and Survivin [5, 6, 7]. This Wnt–YAP crosstalk has been strongly implicated in therapeutic resistance [8, 9, 10, 11, 12]. For instance, in KRAS‐mutant CRC cells, MEK inhibition triggers compensatory activation of the Wnt pathway and induces stemness markers (LGR5, EpCAM, Nanog), which are closely associated with YAP signaling and tumor resistance [8, 9, 10]. Disruption of this oncogenic feedback loop via inhibition of tankyrase (TNKS)—a poly(ADP‐ribose) polymerase that regulates both pathways—has been shown to restore MEK inhibitor sensitivity [13, 14]. By contrast, CRC cells with wild‐type KRAS exhibit reduced reliance on the Wnt–YAP axis and are more responsive to MEK inhibition alone [9, 13]. These observations support the rationale for combinatorial inhibition of both Wnt and YAP pathways in KRAS‐mutant CRC to overcome drug resistance.

TNKS plays a dual role in regulating the Wnt and YAP pathways. It promotes Wnt signaling by poly(ADP‐ribosyl)ating AXIN1/2, thereby targeting them for proteasomal degradation [15, 16]. Simultaneously, TNKS modifies AMOT, a negative regulator of YAP, leading to its degradation and consequent YAP activation [17]. Thus, TNKS inhibition stabilizes both AXIN and AMOT, resulting in the coordinated suppression of Wnt/β‐catenin and YAP signaling. Several TNKS inhibitors, including XAV939, IWR1, G007‐LK, and OM‐153, have demonstrated the ability to stabilize AMOT, downregulate YAP activity, and enhance MEK inhibitor sensitivity in preclinical CRC models [9, 14, 17, 18, 19, 20, 21]. Despite these promising results, the clinical advancement of early‐generation TNKS inhibitors has been limited by dose‐limiting gastrointestinal toxicity [22].

Basroparib (STP1002) is a next‐generation, TNKS‐selective inhibitor that exhibits robust preclinical antitumor efficacy without the toxicity observed with earlier compounds [23]. A Phase I clinical trial (NCT04505839) confirmed its safety and tolerability, supporting its continued clinical development [24]. In our previous study, we demonstrated that basroparib suppresses Wnt‐mediated cancer stemness and restores MEK inhibitor sensitivity in KRAS‐mutant CRC [9]. Building upon these findings, we now show that basroparib also modulates the Hippo–YAP pathway in MEK inhibitor–resistant CRC and other YAP‐driven cancers. Specifically, basroparib suppresses YAP oncogenic activity by stabilizing AMOT, leading to cytoplasmic sequestration of YAP and inhibition of its transcriptional programs. These findings highlight the therapeutic potential of basroparib as a dual‐pathway inhibitor capable of overcoming resistance in Wnt/YAP co‐activated malignancies.

## 2 Materials and methods

### 2.1 Cell culture

All cell lines were obtained from the American Type Culture Collection (ATCC; Manassas, VA, USA) and authenticated by short tandem repeat (STR) profiling according to the 2012 ANSI Standard (ASN‐0002) established by the ATCC Standards Development Organization. The cell lines used in this study include SW480 (ATCC CCL‐228, RRID: CVCL_0546), DLD‐1 (ATCC CCL‐221, RRID: CVCL_0248), SW620 (ATCC CCL‐227, RRID: CVCL_0547), COLO320DM (ATCC CCL‐220, RRID: CVCL_0219), HCT116 (ATCC CCL‐247, RRID: CVCL_0291), RKO (ATCC CRL‐2577, RRID: CVCL_0504), H1299 (ATCC CRL‐5803, RRID: CVCL_0060), BT‐549 (ATCC HTB‐122, RRID: CVCL_1092), MDA‐MB‐231 (ATCC HTB‐26, RRID: CVCL_0062), Capan‐1 (ATCC HTB‐79, RRID: CVCL_0237), H358 (ATCC CRL‐5807, RRID: CVCL_1559), MIA PaCa‐2 (ATCC CRL‐1420, RRID: CVCL_0428), A549 (ATCC CCL‐185, RRID: CVCL_0023), MCF‐7 (ATCC HTB‐22, RRID: CVCL_0031), and H460 (ATCC HTB‐177, RRID: CVCL_0459). COLO320DM, DLD‐1, SW480, SW620, HCT116, H1299, BT‐549, H358, A549, and H460 cells were cultured in RPMI 1640 medium (WELGENE, Gyeongsan‐si, Gyeongsangbuk‐do, Korea). RKO cells were cultured in Eagle's Minimum Essential Medium (EMEM; WELGENE), while MDA‐MB‐231, MIA PaCa‐2, and MCF‐7 cells were maintained in Dulbecco's Modified Eagle's Medium (DMEM; WELGENE). All media were supplemented with 10% fetal bovine serum (FBS; CORNING, Corning, NY, USA), 100 U·mL^−1^ penicillin, and 100 μg·mL^−1^ streptomycin (GenDEPOT, Katy, TX, USA). Cells were incubated in a humidified atmosphere at 37 °C with 5% CO₂. All cell lines were maintained in culture for a maximum of 1.5 months (passages 6–8) and were routinely tested for mycoplasma contamination. All experiments were performed using mycoplasma‐free cells.

### 2.2 Chemical inhibitors

Trametinib was procured from TargetMOL (Wellesley Hills, MA, USA). SCH772984, alpelisib, palbociclib, and paclitaxel were sourced from Selleckchem (Houston, TX, USA). Basroparib was obtained from ST Pharm (Seoul, Korea) [23].

### 2.3 Trametinib‐resistant cell lines

To generate trametinib‐resistant cells, SW480 and SW620 cells were exposed to gradually increasing concentrations of the drug over a 3‐month period, beginning at 1 nm and progressing to 10 nm. Upon establishment of resistance, these cell lines were consistently cultured in 10 nm trametinib (TargetMOL).

### 2.4 Combination index

The combination index was calculated based on the fraction of the effect for each drug combination using the Chou–Talalay method, implemented using the compusyn software (ComboSyn, Inc., Paramus, NJ, USA). A combination index < 1 indicates synergism. The combination index values are categorized as follows: 0.1–0.3 indicates strong synergism, 0.3–0.7 denotes synergism, 0.7–0.9 reflects moderate synergism, 0.9–1.1 suggests a nearly additive effect, 1.1–1.5 indicates moderate antagonism, and values exceeding 1.5 signify antagonism.

### 2.5 Cell viability assay and clonogenic assay

Cell viability and clonogenic assays were conducted as previously reported [9, 23]. Cells were treated with 10 nm trametinib and/or 5 μm basroparib for 72 h. Viability was assessed using WST‐8 reagent (LPS solution; Daejeon, Korea), with absorbance measured at 460 nm using the VersaMax Microplate Reader (Molecular Devices, San Jose, CA, USA). For the clonogenic assay, cells were seeded in triplicate into 60‐mm tissue culture dishes at 1 × 10^3^ cells per dish and were exposed to 5 nm trametinib and/or 5 μm basroparib. After 14 days, the colonies were fixed, stained with 1% crystal violet (MilliporeSigma, Burlington, MA, USA) in 40% methanol, and counted using the ImageJ software (National Institutes of Health, Bethesda, MD, USA).

### 2.6 Transfection and luciferase reporter assay

Transfection and luciferase reporter assays were performed as previously described [9, 23]. SW620 and COLO320DM cells were transiently transfected with Lipofectamine 2000 reagent (Thermo Fisher Scientific, Waltham, MA, USA) and the YAP vector (pQCXIH‐Myc‐YAP‐5SA, addgene, Watertown, MA, USA). For the luciferase reporter assay, CRC cells were transiently transfected with transcriptional enhancer activator domain4 (TEAD4)‐responsive reporter vectors (8xGTIIC‐luciferase, addgene) and phRLuc using Lipofectamine 2000. The assays were conducted using the Dual‐Luciferase® Reporter Assay System (Promega, Madison, WI, USA), and luminescence was measured with a microplate reader (Molecular Devices LLC).

SW620 and trametinib resistant‐SW620 cells were transfected with control and human *AMOT* small interfering RNA (siRNA; 50 nm) using Lipofectamine 2000 reagent (Thermo Fisher Scientific). Cells were assayed 48 h after transfection. Human *AMOT* siRNA (siAMOT) or control siRNA (siCon) was designed by Dharmacon (Lafayette, Co, USA) using the ON‐TARGET plus Human AMOT siRNA‐SMART pool (L‐015417‐01‐0020) or ON‐TARGET plus nontargeting pool (D‐001810‐10‐20).

### 2.7 Western blotting and immunoprecipitation

Western blotting analysis was performed as previously described [23]. Cells were exposed to 10 nm trametinib and 5 μm basroparib for 48 h. Cells were then lysed in RIPA buffer. Proteins were separated by 10% SDS/PAGE and then transferred to PVDF membranes (MilliporeSigma). For immunoprecipitation of endogenous proteins, cell lysates were prepared using NP40 buffer (ELPIS Biotech, Daejeon, Korea). Protein (1 mg) was immunoprecipitated with Protein A agarose beads (GenDEPOT) and the indicated antibody with gentle rotation at 4 **°**C, overnight. Immunoprecipitates were washed four times using NP40 buffer, and bound proteins were dissociated in 50 μL of 2× loading dye. Membranes were incubated with primary antibodies (1 : 1000) overnight at 4 °C and with secondary antibodies (1 : 3000) for 50 min at room temperature. The primary antibodies utilized were YAP (1 : 1000, #14074, RRID:AB_2650491), cysteine‐rich angiogenic inducer 61 (CYR61) (1 : 1000, #14479, RRID:AB_2798492), connective tissue growth factor (CTGF) (1 : 1000, #86641, RRID:AB_2800085), AMOT (1 : 1000, #43130, RRID:AB_2799236), and active β‐catenin (1 : 1000, #8814, RRID:AB_11127203), all sourced from Cell Signaling Technology (Danvers, MA, USA). The β‐actin (1 : 5000, #AM1021B, RRID:AB_2257945) antibody was obtained from Abcam (San Diego, CA, USA), while the phospho‐extracellular signal‐regulated kinase (p‐ERK) (1 : 1000, #sc‐7383, RRID:AB_627545) and ERK (1 : 1000, #sc‐514 302, RRID:AB_2571739) antibodies were acquired from Santa Cruz Biotechnology (Dallas, TX, USA). Detection was conducted using ECL (#RPN2106, Cytiva, Marlborough, MA, USA). Goat anti‐rabbit horseradish peroxidase (#31460, Thermo Fisher Scientific) and goat anti‐mouse horseradish peroxidase (#31430, Thermo Fisher Scientific) were used as the secondary antibodies. Detection was performed using ECL (#32106, Thermo Fisher Scientific) according to the manufacturer's instructions. Western blotting was performed using standard SDS/PAGE protocols. In cases where target proteins differed significantly in molecular weight or gel capacity was limited, the same lysate samples were loaded onto separate gels run in parallel under identical experimental conditions. All blots were processed simultaneously using the same antibodies and exposure settings to ensure consistency.

### 2.8 Immunofluorescence

Immunofluorescence analysis of cells and tumor tissues was conducted as previously described [23]. In summary, cells were fixed in 4% paraformaldehyde (pH 7.4) and permeabilized with 0.1% Triton X‐100 (LPS Solution) in PBS (CORNING) at room temperature. Subsequently, cells were incubated with antibodies against YAP (1 : 200, #14074, RRID:AB_2650491, Cell Signaling Technology) for 2 h. The samples were analyzed using the INCELL analyzer 2200 (Cytiva). For tissue samples, after fixation in 4% paraformaldehyde (pH 7.4), they were incubated in a sucrose solution at 4 °C until fully submerged. The tissues were then embedded in optimal cutting temperature compound and sectioned into 20 μm slices using a cryostat (Leica Biosystems, Wetzlar, Germany). The tissue sections were stained with antibodies against YAP (1 : 100, #376830, RRID:AB_2750899, Santa Cruz Biotechnology), AMOT (1 : 100, #43130, RRID:AB_2799236, Cell Signaling Technology), and Hoechst (1 : 3000, #H3569, RRID:AB_2651133, Thermo Fisher Scientific). Samples were visualized using a confocal microscope (CarlZeiss, Oberkochen, Germany).

### 2.9 qRT–PCR analysis of YAP/TEAD target gene expression

Quantitative polymerase chain reaction (qPCR, LightCycle96, Roche, Mannheim, Germany) was performed using Power SYBR Green PCR Master Mix (Thermo Fisher Scientific) according to the manufacturer's instructions. All results were normalized to glyceraldehyde‐3‐phosphate dehydrogenase levels. The primer sets are presented below.

**Table:** 

CTGF	F	Accgactggaagacacgt
	R	Ccaggtcagcttcgcaag
CYR61	F	Ggtcaaagttaccgggca
	R	Ggaggcatcgaatcccag
AMOT	F	Tctgctcctgctcagactca
	R	Acaggcccatctgttttgtc
AMOTL1	F	Ccacctgagtaccccttcaa
	R	Ggctcgttaccccatactca
AMOTL2	F	Gcttcaatgagggtctgctc
	R	Gaaaacagatggcaccgact

### 2.10 Xenograft model

The xenograft model was established following previously described protocols [9, 23]. In brief, 4‐week‐old female nude mice (CAnN.Cg‐Foxn1 nu/CrlOri) with an average body weight of approximately 20 g, obtained from Orient Bio (Seongnam‐si, Gyeonggi‐do, Korea) were subcutaneously injected with 1 × 10^7^ SW620 cells into their flanks. Mice were anesthetized with a mixture of Zoletil and Rompun (9 : 1, v/v), diluted 10‐fold with saline. To minimize baseline variability, tumor sizes of all mice were measured prior to treatment initiation, and animals were then assigned to experimental groups to ensure comparable mean tumor volumes across groups. Although formal blinding was not implemented during the study, all procedures and analyses were conducted objectively to avoid bias in data interpretation and outcome evaluation. To investigate the antitumor effect of combination therapy, basroparib and/or trametinib were administered orally at doses of 10 mg·kg^−1^ and/or 0.5 mg·kg^−1^ daily, respectively, for 35 days. To establish a trametinib‐resistant SW620 xenograft model, nude mice were injected subcutaneously with 1 × 10^7^ SW620 cells. When the tumors reached approximately 100 mm^3^, the mice were treated daily by oral gavage with trametinib, gradually increasing the dose up to 0.5 mg·kg^−1^ over a period of 4 weeks. As the tumors exhibited regrowth at this time, the mice were then randomly allocated to different treatment groups. To evaluate the efficacy of basroparib in trametinib‐resistant SW620 xenografts, basroparib and/or trametinib were administered orally at 10 and 0.5 mg·kg^−1^ daily for 25 days. All animal procedures, including euthanasia, were conducted in accordance with institutional and national ethical guidelines. Rodent euthanasia was performed using a CO₂ chamber, which recommended a gradual fill method with a displacement rate of 30–70% of the chamber volume per minute to minimize pain and distress.

### 2.11 Cancer cell line encyclopedia and correlation analysis

The Human Protein Atlas dataset (www.proteinatlas.org) was utilized to explore and analyze colorectal cancer RNA‐seq data. This open‐access resource currently provides data for over 5000 tumor samples from 20 different cancer studies. Using this analysis, we ranked 62 colorectal cancer cell lines according to the level of YAP expression. Gene expression data for patient samples from colorectal, breast, lung, and pancreatic cancers were obtained from the TCGA‐Genomic Data Commons via cBioPortal (https://www.cbioportal.org/). The correlations between YAP expression and those of TNKS1 and TNKS2 were assessed using Pearson's correlation coefficient (*r*), which quantifies the linear relationship between two continuous variables. An *r* value approaching 1 indicates a strong positive correlation. Both *r* and the corresponding *P*‐value were calculated using graphpad prism (GraphPad Software Inc, San Diego, CA, USA).

### 2.12 Gene set enrichment analysis (GSEA)

GSEA Preranked was performed using gsea v4.4.0 (Broad Institute). For TCGA tumors, genome‐wide genes were ranked by their Spearman correlation with TNKS1 or TNKS2 expression, and enrichment of the YAP transcriptional signature (CORDENONSI_YAP_CONSERVED_SIGNATURE, MSigDB Human release v2025.1/June 2025, *n* = 57) was examined. Parameters were weighted enrichment statistic, 1000 permutations, and Collapse/Remap to gene symbols = No (input IDs = HUGO gene symbols).

### 2.13 Ethical approval

All animal experiments were performed in accordance with the Animal Protection Act and the Laboratory Animal Act of the Republic of Korea, and institutional guidelines approved by the Korea Institute of Radiological and Medical Sciences (KIRAMS). The study protocol was reviewed and approved by the Institutional Animal Care and Use Committee (IACUC) of the KIRAMS Animal Center (Approval No. KIRAMS2022‐0112), and all procedures complied with relevant ethical regulations and ARRIVE guidelines.

### 2.14 Statistical analysis

All experiments were independently repeated at least three times, and data are expressed as mean ± standard deviation (SD). For comparisons between two independent groups, a two‐tailed Student's *t*‐test was used. For comparisons among multiple groups, one‐way analysis of variance (ANOVA) followed by Bonferroni's *post hoc* test was performed. Statistical significance was defined as *P* < 0.05. *Post hoc* power analyses were conducted using G*Power version 3.1 (Heinrich Heine University, Düsseldorf, Germany; https://download.cnet.com/s/g‐power) to ensure adequate sample size. All statistical analyses were performed using Microsoft Excel and xlstat (Addinsoft, Paris, France; https://www.xlstat.com).

## 3 Results

### 3.1 Basroparib stabilizes AMOT and inhibits YAP‐TEAD signaling in CRC cells

**Fig. 1:**
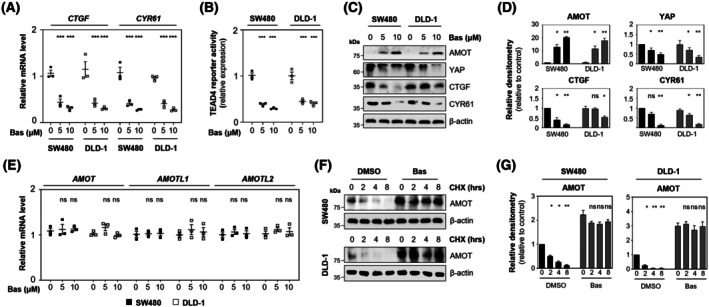
Basroparib stabilizes AMOT and attenuates YAP–TEAD signaling in CRC cells. (A) SW480 and DLD‐1 cells were treated with basroparib (5 or 10 μm) for 48 h. Expression of YAP target genes (CTGF, CYR61) was analyzed by quantitative RT‐PCR (qRT‐PCR). (B) TEAD4 transcriptional activity was measured using a luciferase reporter assay. (C, D) Cells were subjected to immunoblotting with the indicated antibodies, and protein levels were quantified. (E) mRNA levels of AMOT, AMOTL1, and AMOTL2 were evaluated by qRT‐PCR. (F, G) Cells were treated with 5 μm basroparib for 48 h, followed by cycloheximide (CHX, 20 μg·mL^−1^) treatment for the indicated time points prior to immunoblotting. All data are shown as mean ± SD (*n* = 3). Statistical significance was determined by one‐way ANOVA followed by Bonferroni's multiple‐comparisons test. *P < 0.05, **P < 0.01, ***P < 0.001; ns, not significant. AMOT, angiomotin; AMOTL1/2, angiomotin like1/2; Bas, basroparib; CHX, cycloheximide; CRC, colorectal cancer; CTGF, connective tissue growth factor; CYR61, cysteine‐rich angiogenic inducer 61; TEAD, transcriptional enhancer activator domain; YAP, yes‐associated protein.

**Fig. 2:**
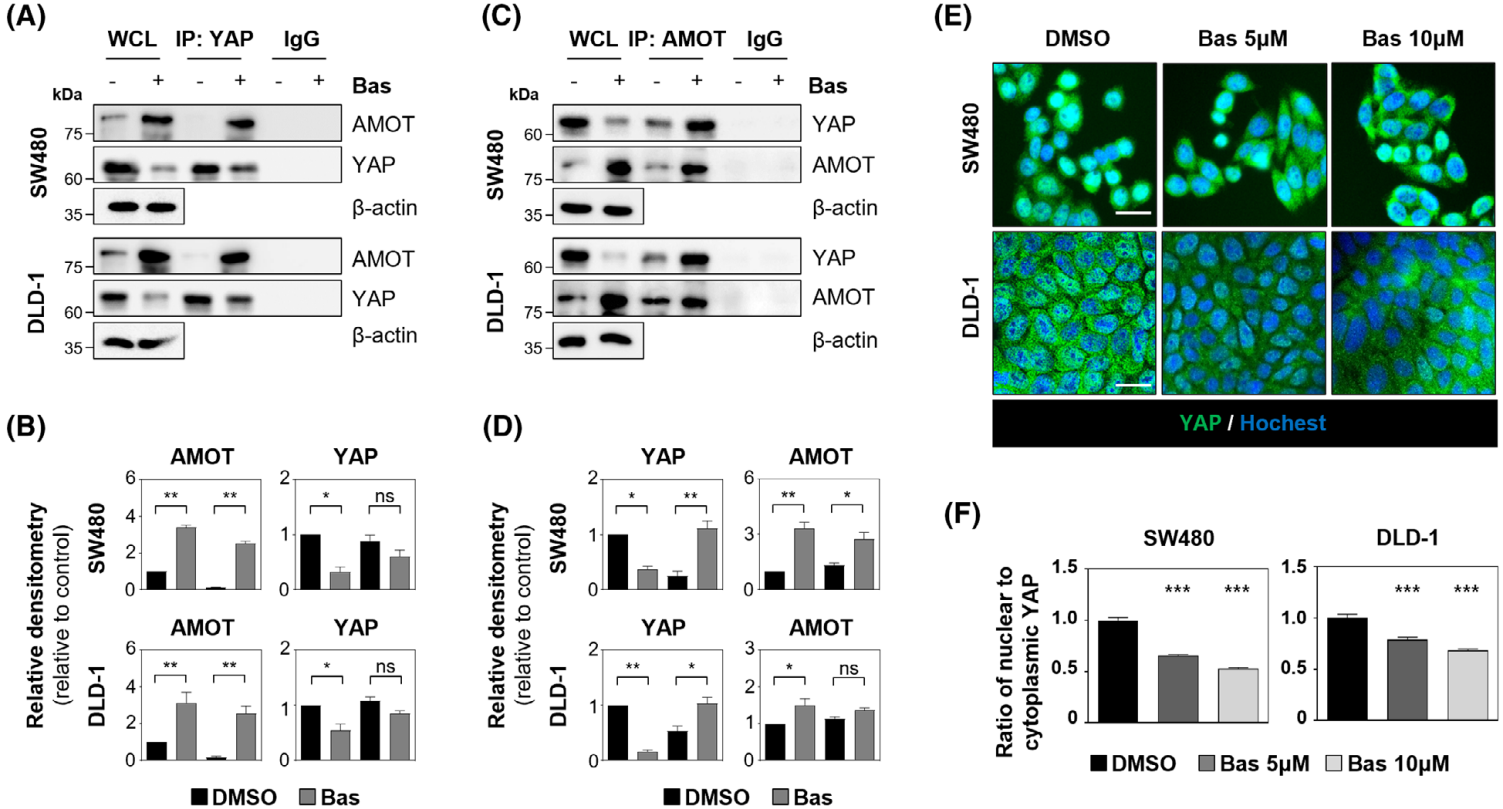
Basroparib promotes YAP–AMOT complex formation and prevents nuclear accumulation of YAP. (A–D) CRC cells were treated with basroparib (5 μm, 48 h), and AMOT–YAP interaction was assessed by co‐immunoprecipitation followed by immunoblotting and quantification. (E, F) Ratio of nuclear to cytoplasmic YAP was evaluated by immunofluorescence staining and quantified. All data are shown as mean ± SD (*n* = 3). Statistical significance in (B and D) was determined using an unpaired two‐tailed Student's *t*‐test, and in (F) by one‐way ANOVA followed by Bonferroni's multiple‐comparisons test. *P < 0.05, **P < 0.01, ***P < 0.001. AMOT, angiomotin; Bas, basroparib; CRC, colorectal cancer; ns, not significant; YAP, yes‐associated protein. Scale bar: 30 μm.

TNKS inhibition is known to stabilize AMOT family proteins, thereby attenuating YAP signaling [17]. To determine whether the TNKS inhibitor basroparib modulates AMOT to inhibit YAP activity, we conducted experiments using SW480 and DLD1 CRC cell lines. Basroparib suppressed YAP signaling in a concentration‐dependent manner, as indicated by reduced expression of YAP target genes and diminished TEAD4‐driven transcriptional activity (Fig. 1A–D). Immunoblot analysis showed decreased levels of YAP, CTGF, and CYR61, alongside a marked increase in AMOT protein levels (Fig. 1B,C). Notably, AMOT mRNA expression remained unchanged following basroparib treatment (Fig. 1E), suggesting a post‐transcriptional mechanism. Consistently, cycloheximide‐chase assays demonstrated that basroparib significantly extended the half‐life of endogenous AMOT proteins (Fig. 1F,G). Co‐immunoprecipitation analysis revealed that basroparib promoted the formation of AMOT–YAP complexes (Fig. 2A–D), coinciding with cytoplasmic translocation of YAP. Immunofluorescence imaging confirmed that basroparib treatment led to a pronounced shift of YAP from the nucleus to the cytoplasm (Fig. 2E,F). This cytoplasmic sequestration is functionally critical, as it prevents YAP from executing its transcriptional coactivator function [17]. Together, these results demonstrate that basroparib inhibits YAP‐driven transcriptional programs by stabilizing AMOT and promoting cytoplasmic retention of YAP, thereby attenuating its oncogenic activity in CRC cells.

### 3.2 Basroparib enhances sensitivity to MEK/ERK inhibitors by inhibiting the YAP pathway in KRAS‐mutated CRC cells

**Fig. 3:**
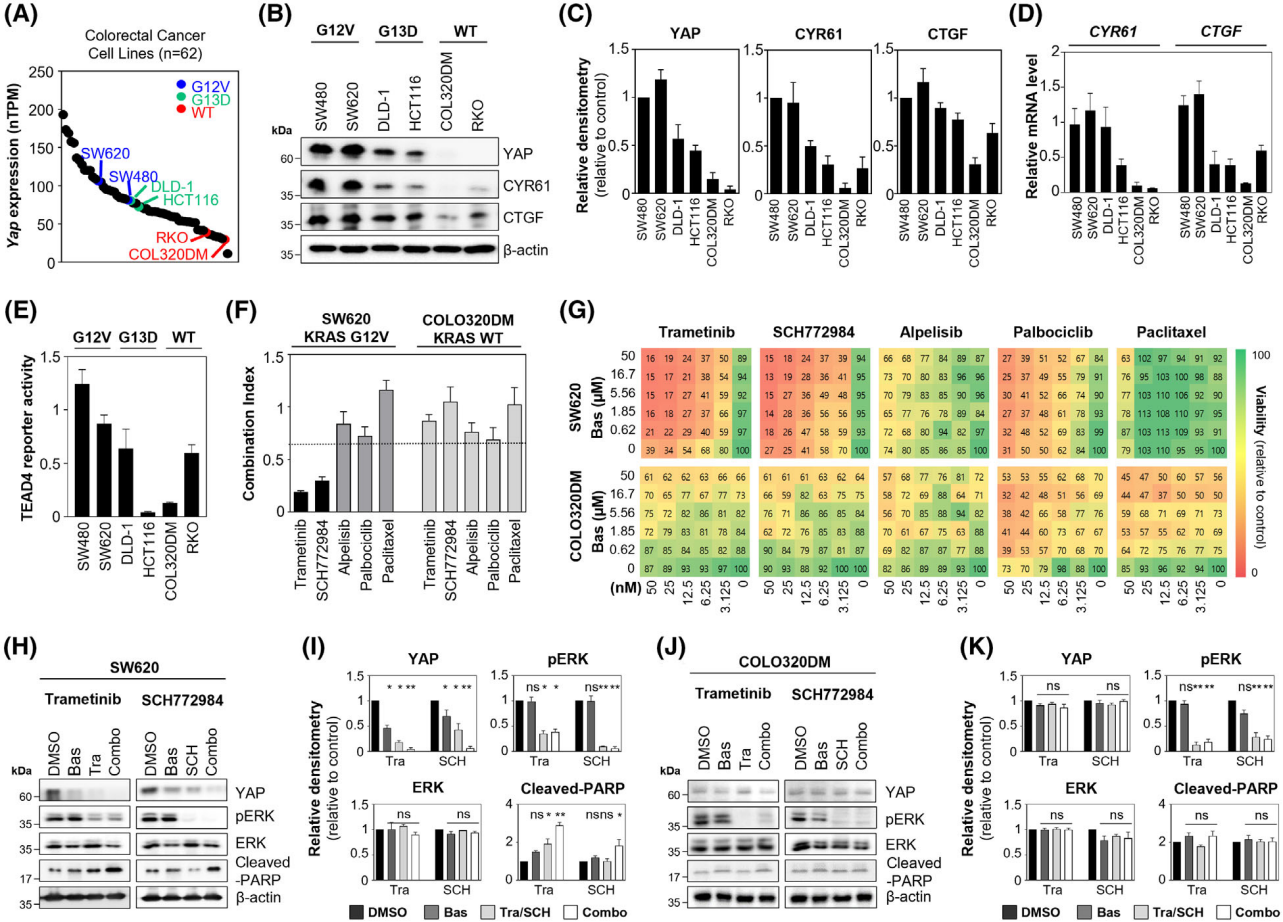
Basroparib sensitizes KRAS‐mutant CRC cells to MEK/ERK inhibition via YAP suppression. (A) YAP expression (nTPM) in CRC cell lines was analyzed using RNA‐seq data from the Human Protein Atlas. (B, C) YAP and its targets (CYR61, CTGF) were analyzed by immunoblotting and quantified. (D) mRNA levels of CYR61 and CTGF were evaluated by qRT‐PCR. (E) TEAD4 reporter activity was measured in KRAS‐mutant and wild‐type CRC cell lines. (F, G) SW620 (KRAS‐mutant) and COLO320DM (KRAS wild‐type) cells were treated with basroparib in combination with trametinib, SCH772984, alpelisib, palbociclib, or paclitaxel for 72 h. CI values were calculated using CompuSyn. Color indicates viability (relative to control), with scale from 0 to 100. (H, I) SW620 and (J, K) COLO320DM cells were treated with basroparib (5 μm) and/or trametinib or SCH772984 (10 nm) for 48 h, followed by immunoblotting and quantification. All data are shown as mean ± SD (*n* = 3). Statistical significance was determined by one‐way ANOVA followed by Bonferroni's multiple‐comparisons test. *P < 0.05, **P < 0.01, ***P < 0.001. Bas, basroparib; CI, combination index; CRC, colorectal cancer; CTGF, connective tissue growth factor; CYR61, cysteine‐rich angiogenic inducer 61; ERK, extracellular signal‐regulated kinase; KRAS, kirsten rat sarcoma; MEK, mitogen‐activated protein kinase; ns, not significant; PARP, Poly (ADP‐ribose) polymerase; SCH, SCH772984; TEAD4, transcriptional enhancer activator domain4; Tra, trametinib; YAP, yes‐associated protein.

Given that basroparib inhibits YAP signaling in CRC cells, we next investigated the relationship between YAP expression and oncogenic drivers, particularly KRAS mutations. Analysis of 62 CRC cell lines from the Cancer Cell Line Encyclopedia dataset revealed a positive association between YAP expression and KRAS mutation status (Fig. 3A). To validate these findings, we selected six CRC cell lines based on their KRAS genotype: wild‐type (COLO320DM and RKO), G12V‐mutated (SW480 and SW620), and G13D‐mutated (DLD‐1 and HCT116). Among these, KRAS G12V‐mutated cells exhibited the highest YAP expression and activity, as evidenced by increased YAP protein levels, upregulation of its transcriptional targets (e.g., CTGF and CYR61), and elevated TEAD4 reporter activity (Fig. 3B–E). Given the established role of YAP in mediating resistance to targeted therapies [3], we examined whether basroparib could sensitize CRC cells to inhibitors of oncogenic pathways, with particular focus on MEK/ERK signaling. To this end, we compared YAP‐high, KRAS G12V‐mutated SW620 cells with YAP‐low, KRAS wild‐type COLO320DM cells. Cells were treated with trametinib (MEK inhibitor), SCH772984 (ERK inhibitor), and alpelisib (PI3Kα inhibitor), as well as palbociclib and paclitaxel to target CDK4/6 and mitotic pathways, respectively. Combination index analyses showed that basroparib markedly enhanced the sensitivity of SW620 cells to MEK and ERK inhibitors, whereas this effect was not observed in COLO320DM cells (Fig. 3F,G). This observation is consistent with a previous report demonstrating similar sensitisation in additional CRC cell lines, including SW480, DLD‐1, and HCT116 [9]. The sensitisation effect of basroparib was selective for MEK/ERK pathway inhibition and not observed with inhibitors of PI3Kα, CDK4/6, or microtubule dynamics. Consistently, co‐treatment with basroparib and either trametinib or SCH772984 led to reduced YAP protein levels in KRAS G12V‐mutated SW620 cells but had no effect in COLO320DM cells (Fig. 3H–K), supporting a KRAS mutation‐dependent mechanism of action. Collectively, these results suggest that basroparib suppresses YAP signaling and enhances the efficacy of MEK/ERK pathway inhibitors specifically in CRC cells harboring KRAS mutations.

### 3.3 Basroparib selectively sensitizes KRAS‐mutated CRC cells to MEK inhibitor in a YAP‐dependent manner.

**Fig. 4:**
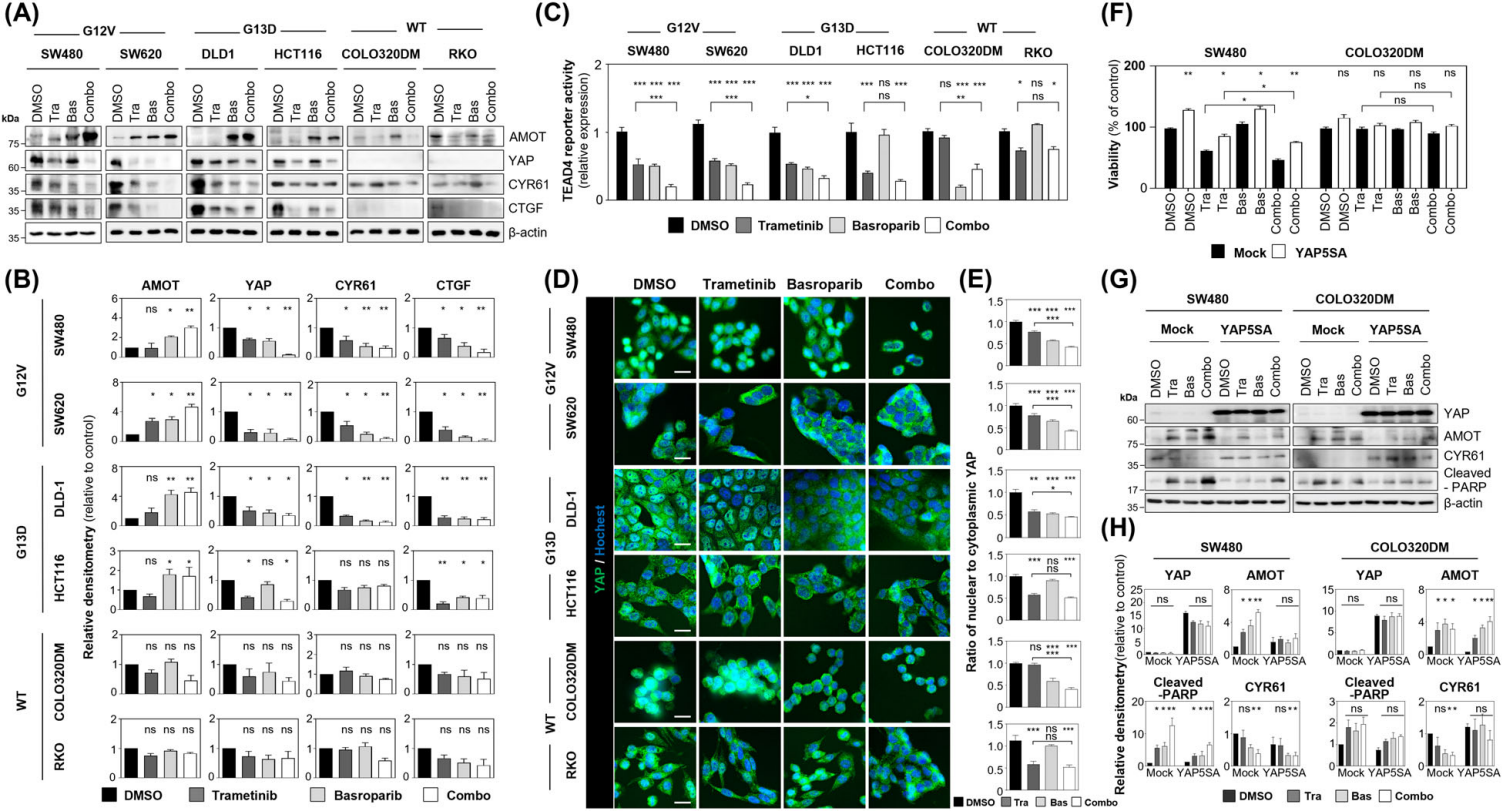
Basroparib‐induced YAP inhibition is KRAS–MAPK dependent. (A, B) KRAS‐mutant and wild‐type CRC cells were analyzed by immunoblotting and quantified. (C) TEAD4 luciferase reporter activity was assessed following treatment with basroparib (5 μm) and/or trametinib (10 nm, 48 h). (D, E) Ratio of nuclear to cytoplasmic YAP was evaluated by immunofluorescence staining and quantified. (F) SW480 and COLO320DM cells were transfected with control or constitutively active YAP5SA. After 24 h, cells were treated with basroparib (5 μm) and/or trametinib (10 nm) for 72 h, and viability was measured. (G, H) Immunoblotting was performed to assess YAP pathway components. SW480 and COLO320DM cells were transfected with control or constitutively active YAP5SA. After 24 h, cells were treated with basroparib (5 μm) and/or trametinib (10 nm) for 48 h. All data are shown as mean ± SD (*n* = 3). Statistical significance in (C, E, and F) was determined using an unpaired two‐tailed Student's *t*‐test, and in (B, C, F, and H) by one‐way ANOVA followed by Bonferroni's multiple‐comparisons test. *P < 0.05, **P < 0.01, ***P < 0.001. AMOT, angiomotin; Bas, basroparib; CRC, colorectal cancer; CTGF, connective tissue growth factor; CYR61, cysteine‐rich angiogenic inducer 61; KRAS, kirsten rat sarcoma; MAPK, Mitogen‐Activated Protein Kinase; ns, not significant; TEAD4, transcriptional enhancer activator domain4; Tra, trametinib; YAP, yes‐associated protein. Scale bar: 30 μm.

To further evaluate whether the synergistic antitumor effects of basroparib and MEK inhibition are mediated through YAP suppression in KRAS‐mutated CRC cells, we examined the impact of combined treatment on YAP signaling. Cotreatment with basroparib and trametinib led to stabilization of AMOT and suppression of YAP signaling specifically in KRAS‐mutated cells. This was supported by reduced expression of YAP and its transcriptional targets, increased AMOT levels (Fig. 4A,B), decreased TEAD4 reporter activity (Fig. 4C), and altered YAP subcellular localization as observed by immunofluorescence analysis (Fig. 4D,E). To functionally confirm the role of YAP in mediating this sensitization, we overexpressed a constitutively active YAP mutant (YAP‐5SA) in KRAS G12V‐mutated SW480 cells. YAP‐5SA overexpression abrogated the enhanced sensitivity to the basroparib–trametinib combination, whereas this effect was not observed in KRAS wild‐type COLO320DM cells (Fig. 4F–H). Together, these findings demonstrate that basroparib enhances the response of KRAS‐mutated CRC cells to MEK inhibition via suppression of YAP signaling, and that this sensitization is YAP‐dependent.

### 3.4 Basroparib enhances sensitivity of YAP‐mediated MEK inhibitor‐resistant CRC by stabilizing AMOT and inhibiting YAP signaling

**Fig. 5:**
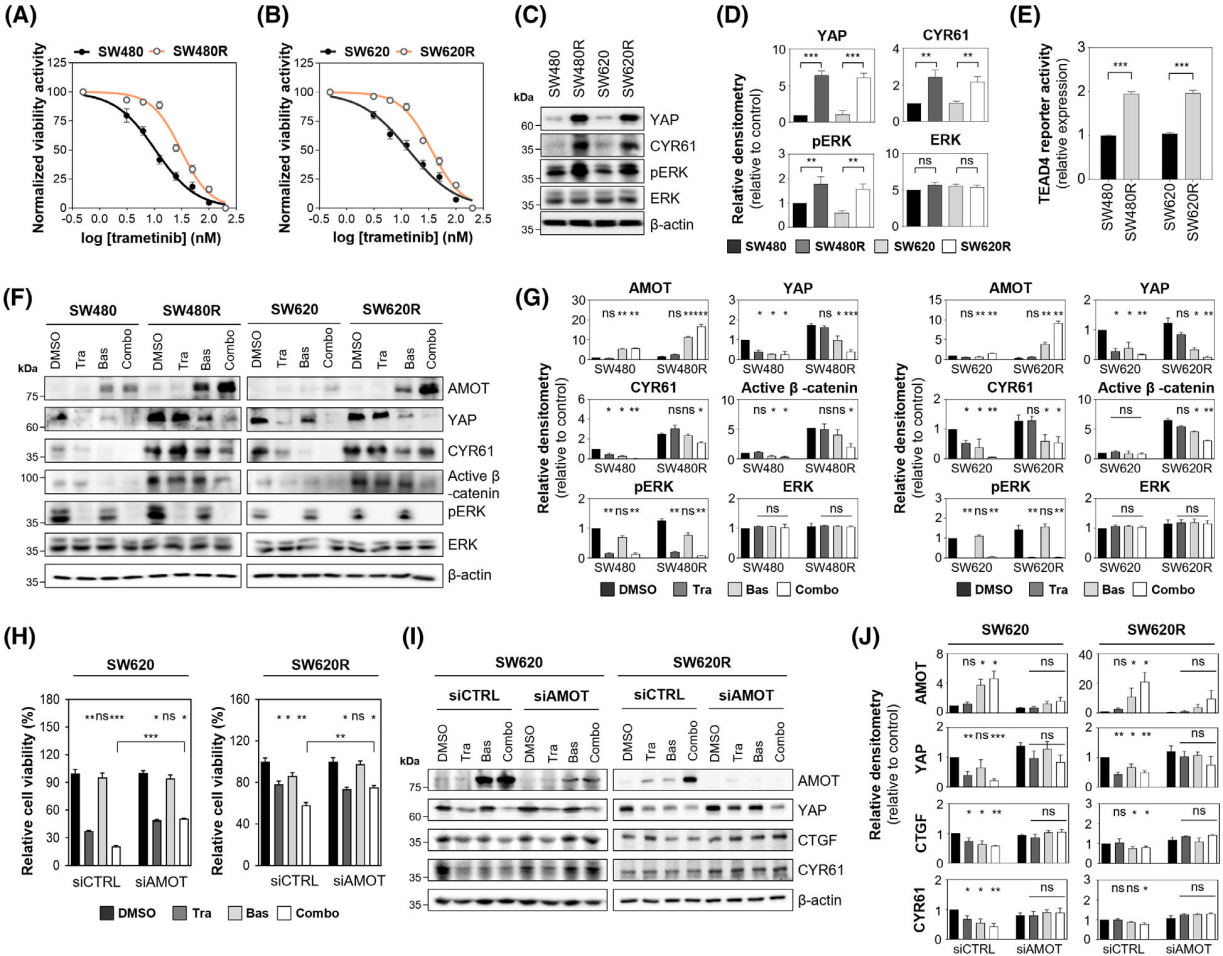
Basroparib overcomes acquired MEK‐inhibitor resistance by modulating the AMOT–YAP axis. (A, B) Dose–response curves of trametinib in parental and trametinib‐resistant CRC cells (3.125–50 nm). (C, D) Immunoblot analysis and quantification of YAP signaling components. (E) TEAD4 luciferase reporter activity in parental and trametinib‐resistant CRC cells. (F, G) Immunoblotting and quantification of AMOT–YAP axis components. (H) Cell viability of SW620 and SW620R cells following siAMOT transfection. (I, J) Immunoblot analysis and quantification of AMOT, YAP, CTGF, and CYR61 protein levels in SW620 and SW620R cells transfected with siAMOT. All data are shown as mean ± SD (*n* = 3). Statistical significance in (A, B, D, E, and H) was determined using an unpaired two‐tailed Student's *t*‐test, and in (G, H, and J) by one‐way ANOVA followed by Bonferroni's multiple‐comparisons test. In panels A and B, *P* < 0.001. *P < 0.05, **P < 0.01, ***P < 0.001. AMOT, angiomotin; Bas, basroparib; CRC, colorectal cancer; CTGF, connective tissue growth factor; CYR61, cysteine‐rich angiogenic inducer 61; ERK, extracellular signal‐regulated kinase; MEK, mitogen‐activated protein kinase kinase; ns, not significant; TEAD4, transcriptional enhancer activator domain4; Tra, trametinib; YAP, yes‐associated protein.

Overexpression or aberrant activation of YAP has been implicated in resistance to MEK inhibitors in multiple cancers, including CRC [2, 3, 4, 20, 25]. To determine whether basroparib could overcome YAP‐mediated resistance in CRC, we generated trametinib‐resistant SW480 and SW620 cell lines by chronically exposing parental cells to increasing concentrations of trametinib. These resistant lines (designated SW480R and SW620R) exhibited significantly elevated IC₅₀ values relative to their parental counterparts, confirming the acquisition of drug resistance (Fig. 5A,B). Importantly, SW480R and SW620R cells displayed increased YAP expression and activity, as indicated by elevated levels of YAP, CYR61, and phosphorylated ERK (p‐ERK) (Fig. 5C,D), as well as enhanced TEAD4 reporter activity (Fig. 5E). These findings are consistent with previous studies implicating YAP activation as a bypass mechanism of resistance to RAF and MEK inhibition [2, 4, 25]. We next investigated whether basroparib could inhibit the Wnt–YAP axis in these resistant cells. Combination treatment with basroparib and trametinib led to AMOT stabilization and a concomitant reduction in both YAP and Wnt pathway activity in SW480R and SW620R cells (Fig. 5F,G). Notably, trametinib alone suppressed p‐ERK but had no significant effect on AMOT or YAP levels in the resistant cells, indicating that basroparib is required to fully disrupt YAP‐associated resistance signaling. Furthermore, AMOT depletion significantly attenuated the sensitisation effect of the combination treatment and restored YAP expression and activity, as evidenced by increased levels of YAP, CTGF, and CYR61 in both SW620 and SW620R cells (Fig. 5H–J), supporting the conclusion that AMOT stabilization is a critical determinant of basroparib‐mediated YAP suppression and MEK re‐sensitisation.

**Fig. 6:**
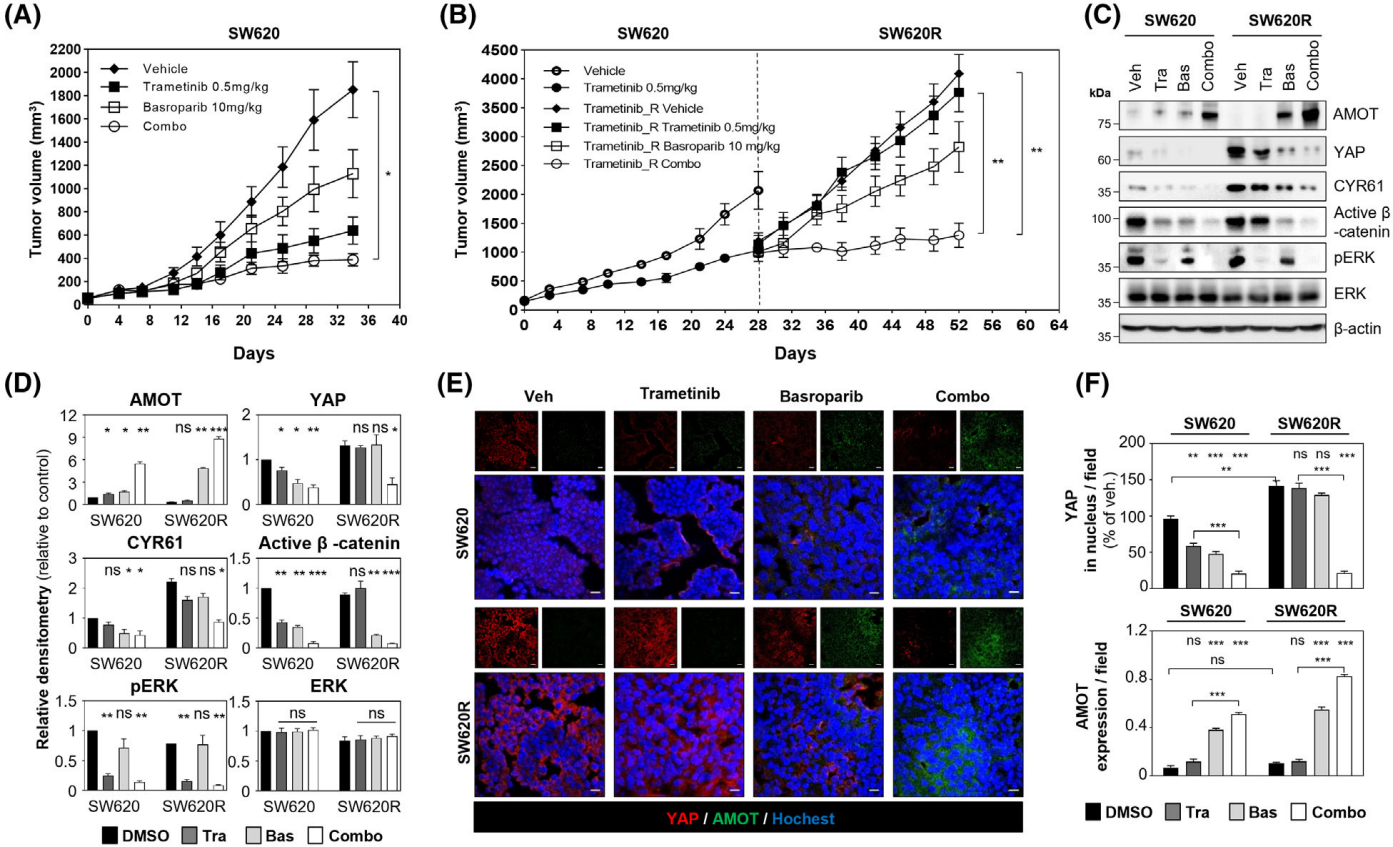
Basroparib inhibits tumor regrowth in trametinib‐resistant SW620 xenografts. (A) Tumor volumes in SW620 xenografts treated orally (PO, QD) with basroparib and/or trametinib (*n* = 8 per group). (B) Tumor volumes in trametinib‐resistant SW620 xenografts treated as above. (C, D) Tumor lysates (*n* = 3 per group) were analyzed by immunoblotting and quantified. (E, F) Tumor sections were stained for YAP and AMOT, and nuclear YAP and total AMOT expression were quantified. All data are shown as mean ± SD (*n* = 3). Statistical significance in (A, B, and F) was determined using an unpaired two‐tailed Student's *t*‐test, and in (D and F) by one‐way ANOVA followed by Bonferroni's multiple‐comparisons test. *P < 0.05, **P < 0.01, ***P < 0.001. AMOT, angiomotin; Bas, basroparib; CYR61, cysteine‐rich angiogenic inducer 61; ERK, extracellular signal‐regulated kinase; ns, not significant; PO, per os (oral); QD, once daily; Tra, trametinib; YAP, yes‐associated protein. Scale bars: 20 μm.

To validate these findings *in vivo*, we employed a KRAS G12V–mutant SW620 xenograft model. Daily oral administration of trametinib (0.5 mg·kg^−1^) or basroparib (10 mg·kg^−1^) alone resulted in tumor growth inhibition (TGI) of 64% and 29.5%, respectively. Notably, combination treatment enhanced TGI to 76%, indicating a synergistic antitumor effect (Fig. 6A). To model acquired resistance *in vivo*, mice bearing SW620 xenografts were treated with trametinib (0.5 mg·kg^−1^) for 4 weeks, resulting in resumed tumor growth despite continuous treatment, thereby establishing a trametinib‐resistant xenograft model. At this stage, trametinib and basroparib monotherapies achieved TGIs of only 7.9% and 31%, respectively. In contrast, combination treatment with basroparib and trametinib significantly suppressed tumor regrowth in the resistant model, achieving a TGI of 68.4% (Fig. 6B). Western blot analysis (Fig. 6C,D) and immunofluorescence (Fig. 6E,F) further confirmed that the combination therapy stabilized AMOT and suppressed YAP‐Wnt axis *in vivo*. These results demonstrate that basroparib reverses YAP‐mediated resistance to MEK inhibition in CRC through AMOT stabilization, both *in vitro* and *in vivo*.

### 3.5 Basroparib sensitizes YAP‐active cancers to MEK inhibitors

**Fig. 7:**
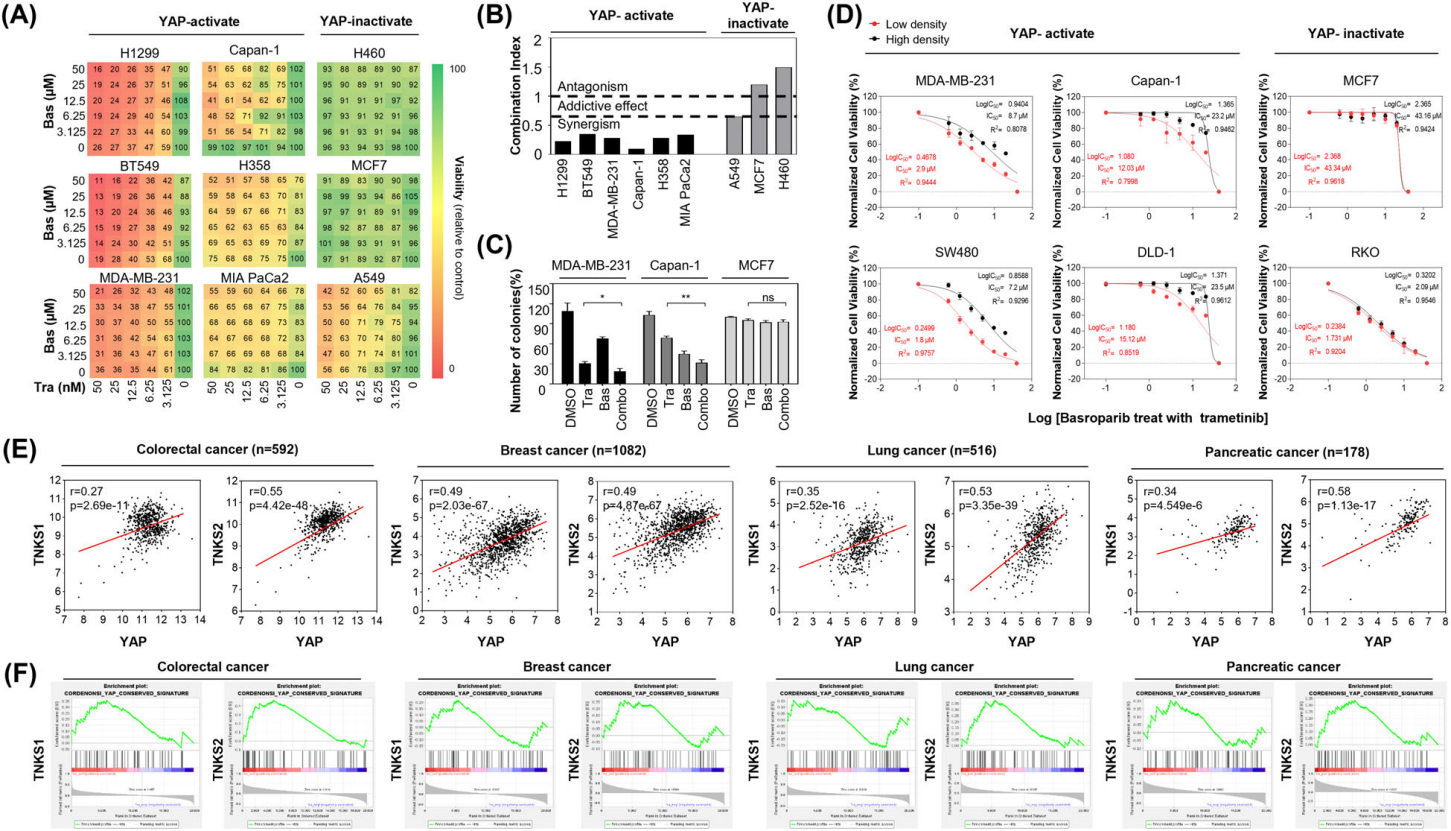
Basroparib potentiates trametinib activity in YAP‐active cancer cell lines. (A, B) YAP‐active and ‐inactive cancer cell lines were treated with increasing doses of basroparib and trametinib for 72 h, and CI values were calculated using CompuSyn. Color indicates viability (relative to control), with a scale from 0 to 100. (C) Clonogenic assays were performed in MDA‐MB‐231, Capan‐1, and MCF7 cells treated with basroparib (5 μm) and trametinib (5 nm). (D) Dose–response curves for the combination treatment in YAP‐active and ‐inactive lines (3.125–50 nm). (E) Correlation between YAP and TNKS1/2 mRNA expression in patient datasets from TCGA (colorectal, *n* = 592; breast, *n* = 1082; lung, *n* = 516; pancreas, *n* = 178). Pearson's *r* and *P*‐values were calculated. (F) GSEA plots for the YAP signature in TNKS1‐ or TNKS2‐ranked gene lists (TCGA). Genes were ranked by Spearman correlation with TNKS expression. The curve shows the running enrichment score; vertical bars indicate the positions of signature members; the dotted line marks the point of maximum deviation. All data are shown as mean ± SD (*n* = 3). Statistical significance in (C and D) was determined using an unpaired two‐tailed Student's *t*‐test. In panel D, *P* < 0.01 in YAP‐active cell lines; ns in YAP‐inactive cell lines. *P < 0.05, **P < 0.01. Bas, basroparib; CI, combination index; GSEA, Gene Set Enrichment Analysis; ns, not significant; TCGA, The Cancer Genome Atlas; Tra, trametinib; YAP, yes‐associated protein.

Building on our findings that basroparib overcomes YAP‐mediated resistance to MEK inhibitors in CRC, we next investigated whether this effect extends to other YAP‐driven malignancies. Based on previously reported classifications [26, 27, 28, 29], we selected a panel of YAP‐active (H1299, BT549, MDA‐MB‐231, Capan‐1, H358, MIA PaCa‐2) and YAP‐inactive (H460, MCF7, A549) cancer cell lines. Combination treatment with basroparib and trametinib selectively sensitized YAP‐active cell lines to MEK inhibition, as demonstrated by combination index analysis (Fig. 7A,B) and reduced colony formation in clonogenic assays (Fig. 7C). In contrast, YAP‐inactive cell lines displayed a minimal response to the combination, supporting a YAP‐dependent mechanism of sensitisation. These results suggest that basroparib may have broad therapeutic utility across a range of YAP‐driven cancers beyond CRC.

Given the known influence of cell density on Hippo–YAP signaling [3], we next assessed whether density affects the responsiveness to this combination therapy. Treatment was conducted under variable seeding densities in YAP‐active (MDA‐MB‐231, Capan‐1, SW480, DLD‐1) and YAP‐inactive (MCF7, RKO) cell lines. Notably, cell viability was more significantly reduced by the combination treatment in YAP‐active cells under high‐density conditions, indicating a density‐dependent therapeutic effect (Fig. 7D). These findings suggest that upstream Hippo pathway regulation may influence the efficacy of TNKS and MEK cotargeting strategies. To further explore the molecular relationship between YAP and TNKS signaling, we conducted Pearson's correlation analyses of YAP and TNKS1/2 expression using patient‐derived tumor datasets. Significant positive correlations were observed across several cancer types, including colorectal (*r* = 0.27, 0.55), breast (*r* = 0.49, 0.49), lung (*r* = 0.35, 0.53), and pancreatic cancers (*r* = 0.34, 0.58) (Fig. 7E). We also performed GSEA, which showed that the YAP transcriptional signature was positively enriched with increasing TNKS1/2 expression in colorectal, breast, and pancreatic cancers (Fig. 7F). These findings support a codependence between YAP and TNKS expression across diverse tumor contexts and further underscore the rationale for dual‐pathway inhibition. Together, these results demonstrate that basroparib sensitizes YAP‐active tumors to MEK inhibition by stabilizing AMOT and suppressing YAP signaling, offering a compelling therapeutic strategy for overcoming resistance in YAP‐driven malignancies.

## 4 Discussion

The Hippo–YAP cascade is now recognized as a central convergence node for tumor progression and resistance to targeted agents, most notably MEK inhibitors. Direct blockade of the YAP–TEAD interface is conceptually attractive, yet the combination of a broad, hydrophobic protein‐binding surface and dose‐limiting albuminuria has thus far prevented clinical translation [3, 4, 25, 30, 31]. Accordingly, recent effort has focused on up‐stream modulators of YAP activity. Among these, TNKS is appealing because its catalytic PARylation targets AXIN1/2 and AMOT—key negative regulators of Wnt/β‐catenin and YAP, respectively. TNKS inhibition therefore offers a single intervention that simultaneously depresses two oncogenic pathways.

Multiple first‐generation TNKS ligands (XAV939, IWR‐1, G007‐LK, OM‐153) validated this dual mechanism *in vitro* [14, 17, 18]; however, dose‐limiting gastrointestinal toxicity curtailed their advancement [32]. Basroparib, a next‐generation, TNKS‐selective inhibitor, overcomes these liabilities: preclinical models show robust antitumour activity without severe intestinal injury [23], and a Phase I study (NCT04505839) confirmed safety at doses up to 360 mg·day^−1^ [24].

Mechanistic interrogation in our study confirms that basroparib stabilizes AMOT, promotes its interaction with YAP, and facilitates the cytoplasmic sequestration of YAP, thereby suppressing YAP‐dependent transcriptional programs [14, 17, 18, 33]. Notably, cotreatment with the MEK inhibitor trametinib further enhanced AMOT stabilization and resulted in more pronounced inhibition of YAP signaling in MEKi‐resistant CRC and other YAP‐active cancer models. These findings suggest that basroparib not only resensitizes Wnt‐driven CRC cells to MEK inhibition but also exhibits broader efficacy in YAP‐driven tumors with intrinsic or acquired resistance to MAPK pathway inhibitors. By reinforcing a physiological tumor‐suppressive mechanism mediated by AMOT [17, 34], basroparib effectively attenuates oncogenic YAP activity and enhances the therapeutic impact of targeted agents. Consistent with this, our AMOT loss‐of‐function experiments demonstrated that depletion of AMOT abolishes basroparib‐mediated YAP inhibition and MEK re‐sensitization, underscoring the central role of AMOT in dictating therapeutic response.

KRAS‐mutant tumors are frequently characterized by elevated YAP signaling, often mediated through the ERK cascade [2, 10, 11, 24]. Paradoxically, MEK inhibition has been shown to induce compensatory activation of YAP, which serves as a bypass resistance mechanism [2, 4, 20, 25]. In our study, KRAS‐mutated and MEK inhibitor‐resistant CRC cells exhibited increased YAP expression and activity. Basroparib effectively resensitised these cells—as well as other YAP‐active models—to MEK inhibition. These findings align with prior reports demonstrating that TNKS inhibition can dismantle resistance‐driving feedback loops by stabilizing AMOT and suppressing nuclear YAP activity, thereby enhancing the efficacy of MAPK‐targeted therapies [10, 13, 35]. Furthermore, transcriptomic analyses revealed a positive correlation between YAP and TNKS1/2 expression across multiple tumor types, including colorectal, breast, lung, and pancreatic cancers. This co‐expression suggests that tumors with high YAP activity may be particularly susceptible to dual inhibition of MEK and TNKS. Recent studies have also implicated YAP/TAZ signaling in resistance to KRAS inhibitors via suppression of pro‐apoptotic genes and activation of the SLC7A5–mTOR axis, thereby sustaining cell survival despite KRAS pathway blockade [36, 37]. In this context, either genetic knockdown of YAP or pharmacologic disruption of the YAP–TEAD complex has been shown to restore drug sensitivity. Given that basroparib suppresses both Wnt and YAP signaling through AMOT stabilization, our findings support its potential as a dual‐pathway inhibitor capable of overcoming resistance to both MEK and KRAS inhibitors.

Importantly, the interplay between Wnt and YAP signaling is not limited to CRC. This functional convergence has been implicated in resistance mechanisms in other malignancies, such as triple‐negative breast cancer (TNBC) and pancreatic ductal adenocarcinoma (PDAC) [11, 12]. In TNBC, YAP activation promotes chemoresistance and the acquisition of stem‐like phenotypes, while in PDAC, YAP contributes to adaptive resistance to KRAS or MEK inhibition. In both cases, TNKS inhibition offers a strategy to concurrently suppress YAP and Wnt activity. Supporting this, our data showed that basroparib sensitized YAP‐active cell lines such as MDA‐MB‐231 (TNBC) and Capan‐1 (PDAC) to MEK inhibition. Mechanistically, this effect was driven by AMOT stabilization, leading to nuclear exclusion of both YAP and β‐catenin and disruption of their oncogenic transcriptional synergy [7]. However, because oncogenic dependencies and YAP regulatory circuitry vary substantially among tumor types, further studies will be necessary to delineate the specific genetic contexts in which basroparib–MEK synergy can be most effectively leveraged.

While direct inhibition of the YAP–TEAD complex remains conceptually appealing, its clinical implementation is hindered by poor druggability and adverse systemic effects [30, 31]. In contrast, upstream targeting of the Hippo–YAP pathway via TNKS inhibition offers a more feasible and potentially safer alternative. Although TNKS enzymes are involved in a variety of cellular processes—including telomere regulation, metabolic control, and Wnt signaling—raising theoretical safety concerns [16, 38], basroparib has thus far demonstrated a favorable pharmacological and toxicity profile in both preclinical and early‐phase clinical settings. Through dual stabilization of AMOT and AXIN, basroparib simultaneously suppresses YAP and Wnt signaling, providing a mechanistically sound and multifaceted strategy to overcome resistance in YAP‐driven cancers.

## 5 Conclusions

In summary, this study identifies basroparib as a potent and selective TNKS inhibitor that suppresses YAP‐driven oncogenic signaling by stabilizing AMOT and facilitating the cytoplasmic sequestration of YAP. By indirectly but mechanistically precise inhibition of YAP activity, basroparib restores sensitivity to MEK inhibitors in KRAS‐mutated and YAP‐active cancers, while concurrently disrupting the Wnt–YAP axis—a central driver of therapeutic resistance. The favorable safety and tolerability profile of basroparib, established in early‐phase clinical trials, further underscores its translational promise. Importantly, basroparib sensitizes a diverse spectrum of YAP‐dependent malignancies—including CRC, TNBC, and PDAC—to MEK inhibition, supporting its broad therapeutic applicability across tumors characterized by YAP hyperactivation. These findings provide strong rationale for the clinical development of basroparib in combination strategies that simultaneously target the Wnt and YAP pathways. Future investigations should focus on identifying predictive biomarkers of response, elucidating the functional contributions of distinct AMOT isoforms, and optimizing combination regimens to maximize therapeutic efficacy while minimizing toxicity in Wnt‐ and YAP‐driven cancers.

## Conflict of interest

Uk‐Il Kim is an employee of ST Pharm Co., Ltd. The remaining authors declare no known competing financial interests or personal relationships that could have appeared to influence the work reported in this paper. The authors declare no potential conflicts of interest with respect to the research, authorship, and/or publication of this article, except as noted below.

## Author contributions

Y‐JK and J‐SK contributed to conceptualization. Y‐JK, U‐IK, and J‐SK contributed to resources. Y‐JK, DYK, YK, and J‐SK contributed to data curation. Y‐JK, DYK, and J‐SK contributed to formal analysis, validation, and investigation. Y‐JK and J‐SK contributed to methodology. Y‐JK, DYK, YK, and J‐SK contributed to visualization. Y‐JK and DYK contributed to writing–draft. U‐IK contributed to writing–review. J‐SK contributed to supervision, funding acquisition, writing–original draft, project administration, and writing–review and editing.

## Data Availability

The authors confirm that the data supporting the findings of this study are available within the article and its supplementary materials, while additional raw or generated datasets used and analyzed during the study can be obtained from the corresponding author upon reasonable request. The datasets have also been deposited in Dryad (https://doi.org/10.5061/dryad.mkkwh71cx).
